# Satisfaction and work overload at Psychosocial Care Centers - Alcohol and Drugs in the Minas Triangle region, Brazil

**DOI:** 10.5327/Z1679443520190434

**Published:** 2019-12-01

**Authors:** Erika Trevisan, Vanderlei José Haas, Sybelle de Souza Castro

**Affiliations:** 1 Department of Occupational Therapy, Universidade Federal do Triângulo Mineiro - Uberaba (MG), Brazil. Universidade Federal do Triângulo Mineiro Department of Occupational Therapy Universidade Federal do Triângulo Mineiro Brazil; 2 Graduate Health Care Program, Universidade Federal do Triângulo Mineiro - Uberaba (MG), Brazil. Universidade Federal do Triângulo Mineiro Graduate Health Care Program Universidade Federal do Triângulo Mineiro Brazil; 3 Department of Collecive Health, Universidade Federal do Triângulo Mineiro - Uberaba (MG), Brazil. Universidade Federal do Triângulo Mineiro Department of Collecive Health Universidade Federal do Triângulo Mineiro Brazil

**Keywords:** mental health, substance-related disorders, mental health services, job satisfaction, outcome assessment

## Abstract

**Background:**

The transformations mental health care underwent in recent years require continuous evaluation of services to guarantee the quality of care. As per the public policies in vigor, care providers are the main technical resource in this field.

**Objective:**

To investigate satisfaction and work overload among workers at Psychosocial Care Centers - Alcohol and Drugs (PCC AD) in the Minas Triangle region, Brazil.

**Methods:**

Participants were 42 care providers at the three PCC AD in the analyzed region. Data collection was performed through a sociodemographic and occupational questionnaire, the Consumers and Caregivers’ Satisfaction with Mental Health Services scale and IMPACT-BR. Numerical variables were subjected to descriptive analysis and expressed as measures of central tendency and dispersion; also Student’s t-test and the non-parametric Mann-Whitney test were used.

**Results:**

The mean score for overall satisfaction was 3.47 and that for general work overload 2.10 on a scale from 1 (lowest) to 5 (highest).

**Conclusion:**

The results point to relevant administrative and operational aspects of work at PCC AD which need to be improved. Improving the staff’s working conditions is essential to ensure high-quality integrated care to individuals with substance abuse.

## RESUMO |

**Introdução:**

O processo de transformação que a saúde mental passou nos últimos anos requer a avaliação contínua dos serviços com o propósito de garantir a qualidade do cuidado. Nessa área, o profissional é o principal recurso técnico para a produção do cuidado em saúde mental, de acordo com as políticas públicas vigentes.

**Objetivo:**

Avaliar a satisfação e a sobrecarga do trabalho nos Centros de Atenção Psicossocial Álcool e Drogas (CAPS AD) da Região do Triângulo Mineiro.

**Métodos:**

Participaram da pesquisa 42 profissionais dos três CAPS AD dessa região. A coleta dos dados foi realizada com questionário sociodemográfico e perfil profissional; escala de avaliação da satisfação dos usuários em serviços de saúde mental (SATIS-BR) e a escala de avaliação do impacto do trabalho em serviços de saúde mental (IMPACT-BR). Foram feitas análises estatísticas descritivas e medidas de centralidade e de dispersão para as variáveis numéricas, bem como testes *t* de Student, não paramétrico de Mann-Whitney e as sintaxes das escalas de pontuação dos testes.

**Resultados:**

A satisfação geral da equipe apresentou escore médio de 3,47, e a sobrecarga geral, escore médio de 2,10, sendo 1 o mínimo e 5 o máximo.

**Conclusão:**

Os resultados apontados no estudo são importantes para identificar os aspectos do trabalho que precisam ser melhorados, sejam eles administrativos, sejam operacionais. Aprimorar as condições de trabalho da equipe técnica é fundamental para a qualidade da atenção integral à saúde das pessoas que fazem uso problemático de álcool e outras drogas.

## INTRODUCTION

Mental health care underwent many and substantial transformations along the past three decades in Brazil. The psychiatric reform movement consolidated significant shifts in the understanding of and approach to mental disorders, including the creation of services alternative to the traditional ones based on social exclusion, violence and chronification of problems.

This process of transformation demands continuous assessment of mental health car, as recommended by the World Health Organization (WHO) to ensure the quality of services, with emphasis on the participation of all three categories of actors: service users, their families and health care providers^1^. Indeed, the need for broad scoped and systematic assessments of health care policies - reinforcing positive aspects, correcting shortcomings and developing new decision-making procedures - is easy to understand. In addition, there seems to be a consensus on that these assessments should involve continuous, systematic and critical-reflexive investigation of practices and processes based on quantitative and/or qualitative indicators. This demands compliance with a minimum of expected standards to obtain relevant and accurate data, i.e. likely to provide valid information on the usefulness of the analyzed practices and procedures, in addition to their feasibility and ethical nature as a function of the focus of research and the peculiarities of national contexts^2^.

The Brazilian Psychosocial Care Centers (PCC) are facilities specialized in mental health care. As part of their prerogatives, PCC are charged of the Psychosocial Care Network (RAPS), care delivery to children and adolescents (infant-juvenile PCC), adults and older adults (PCC I, II, III) and individuals with problems derived from substance abuse (PCC AD)^3^.

While the number of PCC increased considerably in recent years, few studies addressed the relationship between health and work among employees^4^. Knowledge about and understanding psychosocial factors at work to which these workers are exposed might contribute to the development of new perspectives in psychosocial care and occupational health. We call the attention to the particular nature of mental health care, being characterized by subjective practices which demand relativization and individualized approaches, therefore restricting the attempts at objectivization and numerical systematization^5^.

As is known, technology does not have much application in mental health care, but providers, their knowledge and experience are the main resources available^6^. These workers must develop skills to deal with human beings and understand them from the perspective of integrated care within a setting characterized by daily exposure to distress and/or madness, which impregnates the working environment with strong subjective and intersubjective elements and influence their degree of job satisfaction^7^.

As a function of their complexity, the perceptions of mental health care providers have been analyzed from two main perspectives: satisfaction and work overload. For this purpose, the WHO developed the project Consumers and Caregivers’ Satisfaction with Mental Health Services (WHO-SATIS) involving 19 countries to develop measurement scales to assess mental health services^8^.

Satisfaction alludes to pleasant, emotionally positive feelings in regard to the work environment. It depends on personal goals and values and may be influenced by internal and external aspects of the organization of work. Assessing job satisfaction demands considering emotional aspects related to interpersonal relationships among workers, their individual characteristics, values and expectations concerning the work environment and organization^9^.

In turn, work overload involves psychological, emotional and physical issues derived from feelings of pressure, as e.g. excessive job demands, frustration, tiredness, fear of violence and the desire to change jobs^10^. This is a relevant aspect in the assessment of mental health services inasmuch as it has inverse correlation with job satisfaction^4^^,^^11^^,^^12^^,^^13^.

Assessing work overload and satisfaction among PCC AD workers is relevant in the analysis of aspects of the quality of services and to identify work-related factors which interfere with their health. RAPS mental health services are not yet free from obstacles which hinder the attempts at reaching the desired quality, such as insufficient number of facilities, shortage of human and material resources, lack of training and of integration among services. All these factors demand continuous evaluation to correct or minimize flaws which impair the quality of services^1^.

As a function of the aforementioned considerations, the aim of the present study was to assess job satisfaction and work overload among mental healthcare providers at PCC AD in the Minas Triangle region, Brazil.

## METHODS

The present cross-sectional quantitative study was performed at the three PCC AD (1, 2 and 3) available in the Minas Triangle region at the time of data collection. All mid- and high-level employees, to a total of 66, were invited to participate in the study, 42 of whom agreed.

We should observe that it was difficult to raise the awareness of workers, supervisors and managers as to the relevance of participating in studies to investigate significant occupational aspects. The reason was a crisis in the PCC AD system, due to many reasons, at the time of data collection (September through December 2016). We could notice an overall discontent with changes in the operation of services, reduction of the technical personnel, delays in salary payment and political changes in the hierarchical structure of services, among other factors, which contributed to demotivate the workers to participate in the present study. We further call the attention to a historical tradition of neglecting participation in studies that leads to feelings of mistrust and disbelief in regard to the possibility to interfere with the work process.

The instruments administered for data collection were: a sociodemographic and occupational questionnaire and the versions validated for use in Brazil of WHO-SATIS (SATIS-BR) and a scale to assess the impact of work at mental health services (IMPACT-BR)^13^.

STATIS-BR measures the degree of satisfaction of mental health care providers with services. It comprises 30 items distributed across four subscales which assess: satisfaction with the quality of services provided to users, satisfaction with the respondent’s participation within the staff, satisfaction with the working conditions and satisfaction with interpersonal relationships in the workplace. The items are responded on a Likert scale ranging from 1 (very dissatisfied) to 5 (maximum satisfaction). The total score is calculated as the average of the item scores; the higher the score, the higher the level of satisfaction.

In turn, IMPACT-BR measures work overload among mental health care providers. It comprises 18 items distributed across three subscales which assess: impact of work on physical and mental health, effects of work on staff functioning and subjective feelings of being overloaded, and effects of work on the emotional status of workers. There are two additional questions on general issues. The items are responded on a Likert scale ranging from 1 (minimal overload) to 5 (maximum). The total score is calculated as the average of the item scores; the higher the score, the higher the level of work overload^2^^,^^13^.

We first performed descriptive analysis, then we compared the scores on the satisfaction and overload scales to identify the domains with the highest scores by means of Student’s t-test for independent samples in the case of variables with normal distribution and the non-parametric Mann-Whitney test otherwise. The significance level was set to 5%.

The study was approved by the research ethics committee of Universidade Federal do Triângulo Mineiro, ruling no. 1,139,451, Certificate of Presentation for Ethical Appraisal (CAAE) no. 45867315.9.0000.5154.

## RESULTS

From 66 health care providers at the three PCC AD in the Minas Triangle region, 42 agreed to participate in the present study. Most participants were female (81.0%), aged 40 to 49 (31.0%) and had attended non-degree graduate studies (35.7%) or completed higher education (26.2%). The largest proportion of participants corresponded to psychologists (35.7%) and nursing professionals (nurses, nursing technicians and assistants, 21.4%) (Table 1).

**Table 1.** Sociodemographic and educational profile of care providers at Centers of Psychosocial Care - Substance Abuse (PCC AD) in the Minas Triangle region, 2016 (n=42).

Variables	Distribution	
	N=42	%
Sex		
Male	8	19
Female	34	81
Age (years)		
20-29	6	14.3
30-39	8	19
40-49	13	31
50-59	10	24
60-69	1	2.4
Not reported	4	9.3
Educational level		
Incomplete elementary school	1	2.4
Complete elementary school	1	2.4
Incomplete secondary school	3	7.1
Complete secondary school	6	14.3
Incomplete higher education	2	4.8
Complete higher education	11	26.2
Non-degree graduate studies	15	35.7
Master’s degree	3	7.1
Occupation		
Social worker	4	9.5
Nursing assistant	1	2.4
Nurse	4	9.5
Pharmacist	1	2.4
Physician	2	4.8
Psychologist	15	35.7
Nursing technician	4	9.5
Administration technician	3	7.1
Cleaning personnel	2	4.8
Security	2	4.8
Not reported	4	9.5

Most of the sample had 2 to 5 years in the job (38.1%). Most participants rated their academic education insufficient to work at PCC AD (69.0%) even though most had attended training courses less than 6 months (31.0%) or one year (21.4%) earlier. About 52.4% of the sample reported that training was indeed provided at PCC AD, but 78.4% observed they felt they still needed additional training to deal with substance abuse (Table 1).

The average satisfaction score was 3.47, which is indicative of an adequate level. Domain satisfaction with the quality of services exhibited the highest average score (3.72) while satisfaction with the working conditions the lowest (3.18) (Table 2). The internal consistency of SATIS-BR was assessed by means of Cronbach’s alpha, which varied from 0.72 (satisfaction with interpersonal relationships) to 0.87 (overall satisfaction).

**Table 2.** Satisfaction with work at Centers of Psychosocial Care - Substance Abuse (PCC AD), mean, median, standard deviation and Cronbach’s alpha for SATIS-BR global scale and subscale scores, Minas Triangle, 2016 (n=42).

SATIS-BR domains	Mean	Median	Standard deviation	Cronbach’s alpha
Overall satisfaction	3.47	3.46	0.47	0.87
Service quality	3.72	3.63	0.49	0.81
Participation	3.41	3.50	0.75	0.84
Working conditions	3.18	3.11	0.63	0.76
Staff relationship	3.69	3.66	0.80	0.72

Table 3 shows the distribution and comparison of scores on SATIS-BR relative to sex, adequacy of academic education for work at PCC AD and reported need for training. The average score on overall satisfaction was 3.50 for the men and 3.47 for the women, 3.42 for the participants who reported to have adequate training to work at PCC AD and 3.48 for those who did not, 3.49 for those who reported needing additional training and 3.52 for those who did not. None of these differences was statistically significant (p>0.05).

**Table 3.** SATIS-BR scores relative to sex, sufficient academic education and need for training among employees of Centers of Psychosocial Care - Substance Abuse (PCC AD), Minas Triangle, 2016 (n=42).

SATIS-BR domains	Mean	Standard deviation	Mean	Standard deviation	p value*
Sex	Female (n=34)		Male (n=8)		
Overall satisfaction	3.47	0.43	3.50	0.55	0.87
Service quality	3.74	0.46	3.72	0.64	0.93
Participation	3.36	0.73	3.53	0.86	0.63
Working conditions	3.19	0.63	3.16	0.69	0.94
Staff relationship	3.64	0.78	3.79	0.92	0.72
Sufficient academic education	Yes (n=11)		No (n=29)		NR (n=2)
Overall satisfaction	3.42	0.48	3.48	0.48	0.71
Service quality	3.65	0.41	3.76	0.53	0.54
Participation	3.33	0.65	3.41	0.80	0.98
Working conditions	3.14	0.67	3.19	0.64	0.71
Staff relationship	3.43	0.64	3.74	0.86	0.38
Need for training	Yes (n=33)		No (n=5)		NR (n=4)
Overall satisfaction	3.49	0.47	3.52	0.45	0.90
Service quality	3.70	0.49	3.94	0.57	0.34
Participation	3.49	0.73	3.17	2.71	0.33
Working conditions	3.19	0.65	3.31	0.54	0.68
Staff relationship	3.76	0.84	3.33	0.66	0.26

*p<0.05; NR: not reported.

The average work overload score was 2.10. Domain emotional repercussions exhibited the highest average score (2.33) and effects on physical and mental health the lowest (1.94). The internal consistency of IMPACT-BR was assessed by means of Cronbach’s alpha, which varied from 0.52 (staff functioning) to 0.87 (general work overload) (Table 4).

**Table 4.** Work overload among workers at Centers of Psychosocial Care - Substance Abuse (PCC AD) according to IMPACT-BR global scale and subscale scores, Minas Triangle, 2016 (n=42).

IMPACT-BR domains	Mean	Median	Standard deviation	Cronbach’s alpha
General overload	2.10	2.08	0.78	0.91
Physical and mental health	1.94	1.90	0.88	0.65
Staff operation	2.22	2.16	0.84	0.52
Emotional repercussions	2.33	2.40	0.80	0.60

Table 5 shows the distribution and comparison of scores on IMPACT-BR relative to sex, adequacy of academic education for work at PCC AD and reported need for training. The average score on general work overload was 2.11 for the men 1.98 for the women, 2.10 for the participants who reported to have adequate training to work at PCC AD and 2.11 for those who did not, 2.01 for those who reported needing additional training ang and 1.98 for those who did not. None of these differences was statistically significant (p>0.05).

**Table 5.** IMPACT-BR scores relative to sex, sufficient academic education and need for training among employees of Centers of Psychosocial Care - Substance Abuse (PCC AD), Minas Triangle, 2016 (n=42).

IMPACT-BR domains	Mean	Standard deviation	Mean	Standard deviation	p value*
Sex	Female (n=34)		Male (n=8)		
Overall satisfaction	1.98	0.76	2.11	0.79	0.96
Service quality	2.00	0.92	1.67	0.63	0.34
Participation	2.19	0.87	2.35	0.75	0.63
Working conditions	2.33	0.79	2.35	0.91	0.95
Sufficient academic education	Yes (n=11)		No (n=29)		NR (n=2)
Overall satisfaction	1.74	0.64	2.10	0.79	0.29
Service quality	1.63	0.81	1.98	0.86	0.21
Participation	2.07	0.80	2.25	0.88	0.56
Working conditions	2.09	0.80	2.40	0.82	0.29
Need for training	Yes (n=33)		No (n=5)		NR (n=4)
Overall satisfaction	2.01	0.73	1.98	1.05	0.90
Service quality	1.87	0.87	1.68	0.71	0.64
Participation	2.12	0.76	2.16	1.03	0.90
Working conditions	2.31	0.73	2.16	1.32	0.70

*p<0.05; NR: not reported.

## DISCUSSION

The average score on satisfaction was intermediate between indifference and actual satisfaction, being closer to the former. This finding corroborate those of other studies performed with mental health care providers^1^^,^^10^^,^^11^^,^^14^^,^^15^^,^^16^^,^^17^^,^^18^.

A survey of publications on satisfaction and work overload among mental health care providers found that those with the highest level of satisfaction had better physical and mental health, less diseases and higher longevity. The subjective perception of workers may be seen as one of the essential characteristics of the social production of mental health. Workers are the main resource for mental health care, a field in which technology does not have significant application and often lacks operational, social and legal support to ensure integrated care^6^.

In the present study, domain satisfaction with the quality of services exhibited the highest score, close to adequate levels of satisfaction. This finding disagrees from those of some studies, in which overall satisfaction and the other two domains scored higher^1^^,^^8^^,^^16^, but agrees with the results of other studies performed with mental health care providers^9^^,^^14^^,^^19^^,^^20^. Reasons are the particularities of the treatment and care of PCC AD users, the approach to them, the adequacy of services, the level of information afforded on disease and treatment, degree of staff competence and the providers’ understanding of PCC AD users’ problems and expectations.

In one study performed in Paraná, Brazil, with PCC employees, the participants described their job as a source of pleasure based on the service users’ feedback, feelings of satisfaction with the work done and partnership in the organization and execution of activities. All these factors reflect as positive feelings toward work, recognition, gratification and pride with the results of one’s work^21^.

Domain satisfaction with the working conditions exhibited the lowest average score, closer to indifference than to satisfaction. This domain concerns the overall conditions and appearance of facilities, salary and benefits, safety measures, confidentiality and the workplace climate. These findings agree with those of other studies^9^^,^^10^^,^^14^^,^^22^^,^^23^. Working conditions and salary are the main reasons for job dissatisfaction^7^. Several studies also analyzed other factors related to the working conditions, such as shortage of human and material resources, need for proper facilities, lack of materials to conduct workshops, availability of cars for home visits and provision of meals to service users^7^^,^^23^^,^^24^^,^^25^.

A study that surveyed the perceptions and feelings of mental health care providers relative to their practice with focus on job satisfaction evidenced several impasses and conflict in regard to the assumptions underlying the Brazilian psychiatric reform movement which they participants supported. Lack of the resources needed hindered these workers from performing their job as they believe it should be done, which was a cause of distress^26^.

Some of the outstanding factors in regard to work overload include: physical problems, medical visits, medications, effects of work on emotional stability, staff operation, frequently thinking about switching jobs, sick leave due to occupational stress, fear of violence in the workplace, sleep quality, general health, feelings of being overloaded manifested as frustration with outcomes and continuous contact with patients, among others^12^.

Other authors described several obstacles which hinder the work of mental health care providers, such as: frequent interruptions, difficulty to perform different tasks at the same time, impacts of the emotional involvement with patients, unpredictability, lack of essential tools, staff shortage and precarious working conditions. Nevertheless, most studies reported low levels of work overload^1^^,^^8^^,^^11^^,^^17^^,^^20^^,^^27^^,^^28^^,^^29^^,^^30^.

While many mental health care providers are satisfied with their jobs and new practices, work in this field is also a cause of dissatisfaction. Effective psychosocial care must mandatorily consider the mental health of all the involved actors, including the needs, expectations and distress of providers to implement measures to promote satisfaction, happiness and well-being in the workplace. In this regard, the need for greater investment should be acknowledged by municipal managers for workers to be effectively able to interact with service users and the community and thus accomplish the goals of mental health policies^31^.

Domain emotional repercussions of work exhibited the highest score, corresponding to not too much/more or less work overload. This domain also exhibited the highest average scores among the participants who described their academic education as sufficient for work in PCC AD and those who reported needing additional training. These findings agree with those of other studies performed with mental health care providers^1^^,^^4^^,^^8^^,^^17^^,^^27^.

Work in mental health care demands emotional availability from providers beyond the required technical skills. This situation might be a cause of tension, illness and mental imbalance. Our findings point to the relevance of professional training and adaptive strategies, reinforce the need to provide workers adequate assistance and help them develop skills to manage stress and thus reduce their emotional overload.

Care provision to PCC AD workers is essential to ensure the success of treatments as a function of the biopsychosocial complexity of the care required by individuals with substance abuse, including social determinants, peculiar aspects of individuals, their families and life context, patterns of use and individual and collective prejudice. To deliver high-quality integrated care, providers must pay particular attention to their own physical and emotional needs, professional training and strategies to implement a psychosocial care approach that considers social relationships and emotional, affective and biological aspects, consequently challenging in actual practice and demanding interdisciplinary approaches.

Domain physical and mental overload exhibited the lowest average score, close to the level of not too much work overload. This domain comprises five items: frequency of physical problems, frequency of medical visits, frequency of medication use, effects of work on emotional stability and need of professional care^13^. Our findings agree with those of other authors^1^^,^^4^^,^^8^^,^^17^^,^^20^^,^^28^^,^^29^^,^^31^ and suggest that the impact of work might be compensated by other factors, such as subjective commitment to work, ability to develop subjective strategies to perform tasks and staff relationships^1^.

## CONCLUSION

The results of the present study indicate that the participants were satisfied with their job, especially as concerns the care provided to service users, staff relationships, participation in decision making, professional competence and understanding of users’ problems and expectations. These findings evidence the participants’ commitment to integrated health care, RAPS development and the psychiatric reform movement.

Factors associated with work overload mainly concerned institutional obstacles, particularly the excessive bureaucracy of public administration, shortage of human and material resources, low salary and low adherence of service users to treatment. All these conditions are a cause of job dissatisfaction and work overload. The results obtained in the present study might represent an important contribution to the improvement of the quality of service delivery, as well as to the care of mental health care providers. Additional studies are needed to identify other administrative and operational aspects of the job of this occupational group which still need to be improved.

Given that providers are the main resource in psychosocial care, improving their working conditions is essential to ensure high-quality and integrated care to individuals with substance abuse. These improvements concern job stability, adequate salary and career plans including promotions, better facilities in terms of comfort, appearance and safety, theoretical and practical training and supervision vis-à-vis actual challenges in everyday practice and the workplace climate. Scientific, technical and financial investment is need for these changes to occur, while their effects will reflect on the care provided to service users and their families.

In the present study, we identified reasons for job dissatisfaction and work overload which need to be discussed and thought over by PCC AD workers and managers to ensure integrated health care to individuals with substance abuse.
